# aPKCζ-dependent Repression of Yap is Necessary for Functional Restoration of Irradiated Salivary Glands with IGF-1

**DOI:** 10.1038/s41598-018-24678-4

**Published:** 2018-04-20

**Authors:** Alejandro M. Chibly, Wen Yu Wong, Maricela Pier, Hongqiang Cheng, Yongxin Mu, Ju Chen, Sourav Ghosh, Kirsten H. Limesand

**Affiliations:** 1 The University of Arizona, Cancer Biology Graduate Program, Tucson, AZ 85721 USA; 2 Yale University, Department of Neurology, New Haven, CT 06511 USA; 3 The University of Arizona, Department of Nutritional Sciences, Tucson, AZ 85721 USA; 4 University of California San Diego, School of Medicine, San Diego, CA 92093 USA

## Abstract

Xerostomia and salivary hypofunction often result as a consequence of radiation therapy for head and neck cancers, which are diagnosed in roughly 60,000 individuals every year in the U.S. Due to the lack of effective treatments for radiation-induced salivary hypofunction, stem cell-based therapies have been suggested to regenerate the irradiated salivary glands. Pharmacologically, restoration of salivary gland function has been accomplished in mice by administering IGF-1 shortly after radiation treatment, but it is not known if salivary stem and progenitor cells play a role. We show that radiation inactivates aPKCζ and promotes nuclear redistribution of Yap in a population of label-retaining cells in the acinar compartment of the parotid gland (PG)– which comprises a heterogeneous pool of salivary progenitors. Administration of IGF-1 post-radiation maintains activation of aPKCζ and partially rescues Yap’s cellular localization in label retaining cells, while restoring salivary function. Finally, IGF-1 fails to restore saliva production in mice lacking aPKCζ, demonstrating the importance of the kinase as a potential therapeutic target.

## Introduction

Xerostomia and loss of saliva are common side effects of radiotherapy in individuals undergoing treatment for head and neck cancer. Though there is no cure, salivary function has been partially restored in animal studies by means of transplantation^1,2^, gene therapy^3,4^, and pharmacological intervention^5–7^. In humans, a phase I clinical trial using adenoviral-based delivery of aquaporin 1 gene (AdhAQP1) showed improvements in saliva production in five out of eleven patients^8^. Studies have also suggested therapeutic potential in progenitor cells found in the developing salivary glands of mice, such as Keratin 5 (K5), Keratin 14 (K14), and c-kit-expressing progenitors^1,2^. However, it is unknown whether radiation disrupts the stem or progenitor cells in the adult salivary glands, and whether these endogenous cells can be stimulated post-therapy to restore saliva. A drawback in trying to answer this question is the lack of specific markers to delineate the diverse nature of salivary gland stem cells, particularly in the adult PG, which is thought to be the most radiosensitive. Our group previously identified a heterogeneous population of label-retaining cells (LRCs) in the salivary glands, which co-localized with known progenitor markers, such as K5 and K14^9^. Interestingly, many of these cells are found in the acinar compartment of the gland, which has been shown to replenish itself during homeostasis and is thought to contribute to gland regeneration^10^. Label retaining cells are present for at least 30 days post-radiation^9^, but function of the salivary glands is not restored^7,11,12^. Understanding the mechanisms that prevent LRCs from repairing irradiated salivary glands will be instrumental in designing therapies to target specific populations of endogenous cells to promote wound healing without the need for transplantation.

Regarding pharmacological stimulation of specific salivary SPCs, administration of Glial cell line–derived Neurotrophic Factor (GDNF) 1-day post-radiation rescued the submandibular gland (SMG) from radiation-induced damage in mice. The effect of GDNF likely involved expansion of the Lin^−^CD24^+^c-Kit^+^Sca1^+^ subpopulation of progenitor cells, in which the receptor for GDNF is enriched^5^. In a different study, neurturin (NRTN) was shown to promote epithelial regeneration in irradiated SMG explants from mouse embryos^13^. Regeneration was mediated by the protective effect of neurturin on parasympathetic nerves, which in turn promotes survival of Keratin-5 progenitors^14^. Both GDNF and NRTN were administered during acute stages of the glandular response to radiation injury. In the case of GDNF, which was administered *in vivo*, this corresponds to a stage in which chronic loss of function has not yet developed^15^. This is problematic when targeting specific cell populations, such as Lin^−^CD24^+^c-Kit^+^Sca1^+^, because whether they can be stimulated at later stages of radiation-induced damage is unknown. Finally, previous work from our lab showed that systemic administration of Insulin-like growth factor 1 (IGF-1) at 4 to 7 days post-radiation, after acute loss of function is observed, effectively restores saliva production in mice^7^; however, whether IGF-1-induced recovery of function involves stimulation of endogenous salivary SPCs has not been studied.

SPCs undergo compensatory proliferation to promote tissue regeneration following injury^16,17^. This process requires signals from the microenvironment, apoptotic, and inflammatory cells^18–22^. Additionally, the choice of which cell fate decision to take – to expand the pool of stem cells or to differentiate into a more specialized cell – is regulated by complex interactions between SPCs and their niche^22,23^, via adherens junctions, tight junctions, and apical-basal polarity complexes (Par-aPKC, Crumbs 3, and Scribble)^24–26^. Interestingly, the Par3-aPKC complex, which is comprised of atypical protein kinase C zeta or iota (aPKCζ, aPKCι), partitioning defective 3 (PAR3) and 6 (PAR6), and cell division control 42 (CDC42), is involved in appropriate formation of the apical-lateral membrane borders^27,28^, as well as regulating cell division and cell fate in lower organisms and mammalian cells^21,29–32^. Disruption of this complex has been linked to deficient wound healing^30,31,33,34^. Another important player in regulating function of SPCs during tissue repair is the transcriptional co-activator Yap, the main effector downstream of Hippo pathway, involved in orchestrating proper organ formation^35,36^. In the embryonic SMG, increased levels of Yap and its paralog Taz, were associated with assembly of tight junctions during the process of branching^37^. Interestingly, IGF-1, a growth factor previously shown to restore function of irradiated salivary glands in mice^7^, has been shown to induce activation of aPKCζ^38,39^, and aPKCζ is a known negative regulator of Yap during tissue regeneration^40^. Thus, determining whether these pathways are regulated in LRCs by IGF-1 during functional restoration of salivary glands is warranted to develop novel regenerative therapies for salivary gland dysfunction and xerostomia.

Here, we show that radiation injury promotes compensatory proliferation in a population of LRCs in the acinar compartment of irradiated PG. Remarkably, the initiation of this proliferative program coincides with inactivation of aPKCζ and increased nuclear localization of the transcriptional coactivator Yap. Further, we demonstrate that aPKCζ is required to restore function of irradiated salivary glands using IGF-1. This restoration process involves maintenance of aPKCζ phosphorylation and modulation of nuclear translocation of Yap in acinar LRCs in an aPKCζ-dependent fashion.

## Results

### Radiation induces compensatory proliferation of LRCs in the acinar compartment of Parotid Gland

**Figure 1:**
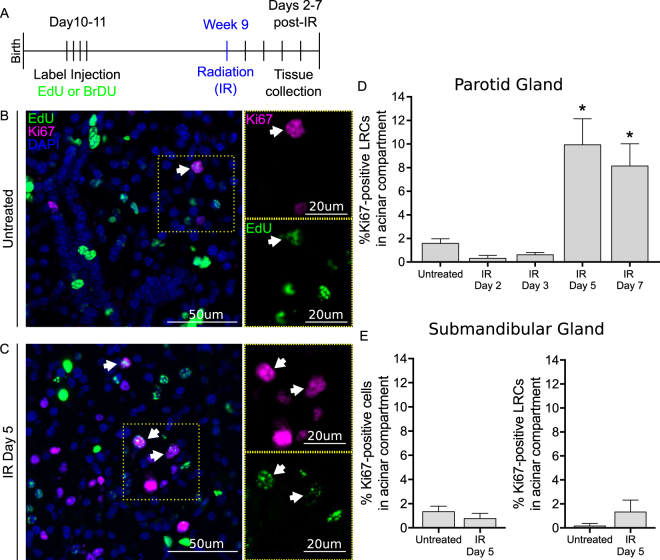
Acinar LRCs undergo compensatory proliferation 5–7 days post-radiation. (**A**) Label-retaining assay: 10-day old FVB mice received four EdU (100 mg/kg) or BrdU (30 mg/kg) injections 12-hours apart (n ≥ 3 for all groups). At 9 weeks of age, mice received a single 5 Gy dose of radiation to the head and neck and PGs were collected at days 2–7 following treatment for analysis. Representative images of control and irradiated PGs immunostained for EdU (Green) and Ki67 (Magenta) are shown in panels B and C, respectively. Proliferative LRCs are shown with a white arrow. (**D**) Percentage of Ki67-positive LRCs in acinar compartment in PG. Statistical differences vs untreated group are represented by a star (p < 0.05 by one-way ANOVA with follow up Tukey’s multiple comparisons test). (**E**) Percentage of Ki67-positive cells in acinar compartment, as well as Ki67-positive acinar LRCs in acinar compartment of SMG.

Radiation induces compensatory proliferation in salivary glands 6–9 days post-treatment^7,41^, presumably to attempt to restore function, but it’s unclear whether salivary SPCs play a role. We hypothesized that LRCs, which express markers of known salivary progenitors^9^, participate in this response. LRCs were identified using the BrdU or EdU label-retaining assay as previously described (Fig. 1A and ref.^9^). To determine whether LRCs undergo radiation-induced compensatory proliferation, tissue sections of parotid and SMG were immunostained for the proliferation marker Ki67 in combination with a marker for the LRCs (Fig. 1B,C). Ki67-positive LRCs were quantified within the acinar-enriched or ductal compartments at days 2 to 7 after radiation treatment (Fig. 1A,D, Fig. S1A, See methods for description of each compartment).

In PGs, proliferation of non-irradiated (untreated) LRCs from the acinar-enriched compartment (herein referred to as acinar LRCs) is 1.58% and increases at days 5 and 7 after radiation treatment (9.93% and 8.14%, respectively, p < 0.05 Fig. 1D). The percentage of Ki67-positive acinar LRCs at day 4 post-radiation is not different than untreated acinar LRCs (data not shown), indicating that compensatory proliferation of acinar LRCs started at day 5. Notably, LRCs are only a portion of the proliferative population. Non-LRCs in the acinar-enriched compartment also show an increase in proliferation at days 5 and 7 post-radiation (9.87% and 8.55%, respectively p < 0.05) in comparison with untreated controls (1.68%, Fig. S1B). In contrast, ductal LRCs do not proliferate and proliferation of non-LRCs in the ductal compartment is not different from untreated controls at the evaluated time points (Fig. S1C). In contrast with PGs, radiation did not increase proliferation in SMG at day 5 post-treatment (Fig. 1E). Since initiation of compensatory proliferation did not involve activity of the ductal compartment, further analysis is focused exclusively on acinar LRCs.

### Loss of aPKCζ induces a hyper-proliferative phenotype and promotes nuclear translocation of Yap in acinar LRCs

**Figure 2:**
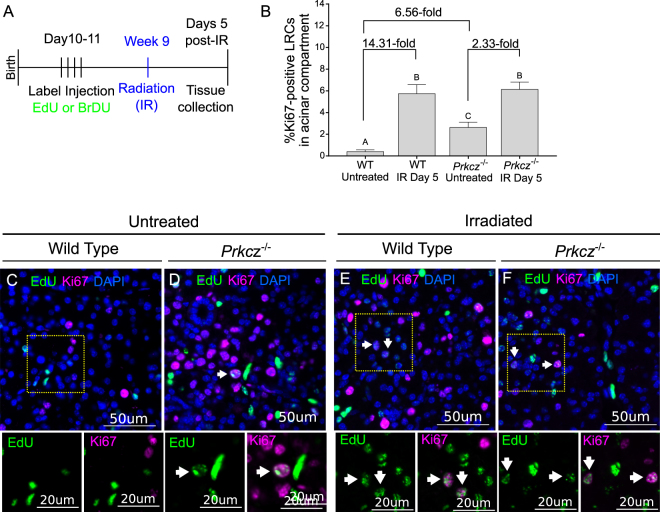
aPKCζ inhibits proliferation of LRCs in acinar compartment. (**A**) Experiment setup. Label-retaining assay was performed in *Prkcz*^*−/−*^ and wild-type (C57BL/6 J) mice as described in methods section (n ≥ 3 for all groups). PGs were collected 5 days following radiation treatment for analysis. (**B**) Percentage of Ki67-positive LRCs in acinar compartment of *Prkcz*^*−/−*^ and wild-type mice. Representative images of control and irradiated PGs immunostained for EdU (Green) and Ki67 (Magenta) are shown in panels C–F. Proliferative LRCs are shown with a white arrow. Different letters represent statistical differences between groups (p < 0.05 by one-way ANOVA with follow up Tukey’s multiple comparisons test).

Cell polarity, and aPKCζ specifically, are known regulators of SPC proliferation^21,30,33,34,40^; thus, we hypothesized that aPKCζ regulates radiation-induced compensatory proliferation of acinar LRCs. Because current aPKCζ inhibitors are not suitable for dosing *in vivo*^42^, and they display non-specific inhibition of aPKCι - an isoform of the kinase that has been reported to have differing functions^43^, we tested our hypothesis by measuring proliferation of LRCs in a knockout mouse model for the *Prkcz* gene (*Prkcz*^*−/−*^, Fig. 2A–F, Fig. S2). Baseline levels of acinar LRC proliferation are 6.56-fold higher in non-irradiated *Prkcz*^*−/−*^ glands compared to wild type controls (Fig. 2B–D, p < 0.05), indicating that aPKCζ suppresses proliferation of acinar LRCs during glandular homeostasis. Consistent with previous experiments, irradiated glands show a 14.31-fold increase in proliferation of wild type acinar LRCs (WT LRCs) at day 5 post-radiation (Fig. 2B, p < 0.05). In irradiated *Prkcz*^*−/−*^ acinar LRCs (KO LRCs), proliferation at day 5 post-radiation is 2.33-fold higher when compared to their respective unirradiated controls (p < 0.05, Fig. 2B). Proliferation in irradiated KO LRCs is not different from irradiated WT LRCs (p = 0.96). These data combined suggest that radiation-induced compensatory proliferation is partially aPKCζ-dependent, whereas the remaining increase (the 2.33 fold-increase in irradiated KO LRCs) is likely downstream of different radiation targets (Fig. 2B). Alternatively, our data may also point at a significant role of aPKCζ in regulating proliferation of LRCs in physiological conditions that is independent of radiation-induced compensatory proliferation.

**Figure 3:**
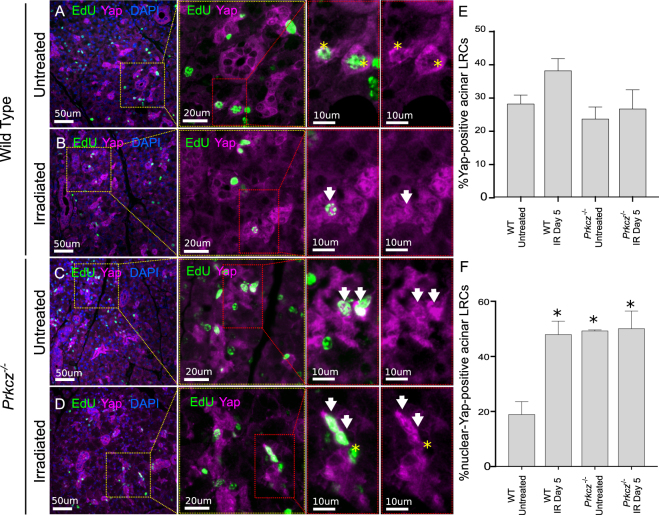
Radiation promotes nuclear translocation of Yap in acinar LRCs downstream of aPKCζ. Immunostaining for Yap (magenta) and EdU (green) of PGs from wild type (C57BL/6 J) and *Prkcz*^*−/−*^ mice are shown in panels A–D. White arrows point at Yap-positive LRCs with nuclear Yap. Yellow stars indicate LRCs with membrane/cytoplasmic Yap. (**E**) Quantification of the percentage of Yap*-*positive acinar LRCs. (**F**) Quantification of the percentage of Nuclear-Yap + LRCs in acinar compartment (white arrows). At least 3 mice were used for each group. One-way ANOVA followed by Tukey’s multiple comparisons test was performed. Stars represent statistical differences vs wild type untreated group (p < 0.05).

It is known that the Hippo pathway and aPKCζ signaling interact to modulate proliferation during homeostasis^24^. Additionally, Yap has been reported to act downstream of aPKCζ in regulating stem cell function^40^ and to drive intestinal regeneration following injury^44^. In non-irradiated salivary glands, Yap is found in both acinar and ductal compartments and is predominantly cytoplasmic. To determine if aPKCζ regulates Yap in the PG after radiation injury, we measured protein and gene expression levels of Yap in whole gland homogenates from untreated and irradiated *Prkcz*^*−/−*^ and WT mice (Fig. 3). Yap protein levels (Fig. S3A) and gene expression (Fig. S3B) are not different in Prkcz^*−/−*^ mice compared to WT controls, nor in irradiated glands compared to untreated samples, indicating that aPKCζ does not affect Yap expression in salivary glands. Because LRCs represent a small fraction of cells in PG, it is possible that changes in Yap wouldn’t be reflected in whole gland homogenates. Thus, localization of Yap was evaluated specifically in acinar LRCs by immunofluorescence analysis (Fig. 3A,D). Since Yap’s transcriptional activity occurs when Yap translocates to the nucleus, nuclear localization of Yap was also quantified. The percentage of WT LRCs that show positive staining for total Yap (both nuclear and cytoplasmic) did not change at day 5 following radiation (Fig. 3E, p = 0.27), when compensatory proliferation is initiated in this population. Interestingly, nuclear localization of Yap is increased in irradiated WT LRCs (p < 0.005, Fig. 3B,F, white arrow). In a similar fashion, the percentage of KO LRCs with total Yap is not different in unirradiated mice compared to wild type controls (p = 0.93, Fig. 3C,E), and remains unchanged 5 days after radiation (p = 0.99, Fig. 3D,E). In contrast, nuclear Yap is highly increased in non-irradiated and irradiated KO LRCs (p < 0.001, Fig. 3C,D,F: White arrows), compared to WT LRCs. The percentage of nuclear Yap in untreated KO LRCs is similar to nuclear Yap in irradiated WT LRCs (p > 0.90, Fig. 3F). Of note, cytoplasmic Yap is detected in virtually all Yap + LRCs regardless of nuclear localization, and the percentage of LRCs with cytoplasmic Yap did not change with radiation or genetic ablation of *Prkcz* (Fig. 3E).

In contrast with our observations in parotid gland, the percentage of LRCs with nuclear Yap is not different in irradiated SMGs compared to control tissues (p = 0.95, Fig. S4). Thus, further analysis is performed only in parotid glands. Taken together, these results suggest that radiation promotes nuclear localization of Yap in acinar LRCs of the parotid gland during initiation of compensatory proliferation, without global changes in Yap expression. Furthermore, aPKCζ inhibits nuclear Yap but it does not affect the global protein and gene expression levels of Yap in PGs.

### Post-therapeutic administration of IGF-1 results in maintenance of aPKCζ phosphorylation and reduces nuclear localization of Yap in irradiated LRCs

Genetic ablation of *Prkcz* demonstrated that aPKCζ inhibits proliferation of acinar LRCs and decreases the percentage of acinar LRCs with nuclear Yap; however, it does not address the question of whether radiation injury disrupts aPKCζ activation in acinar LRCs specifically. In addition, previous data from our lab showed that systemic post-therapeutic administration of IGF-1 restores saliva production in irradiated mice and inhibits radiation-induced compensatory proliferation^7^. Therefore, to test whether activation of aPKCζ is involved in functional restoration of irradiated salivary glands, we also analyzed PGs from IGF-treated mice.

**Figure 4:**
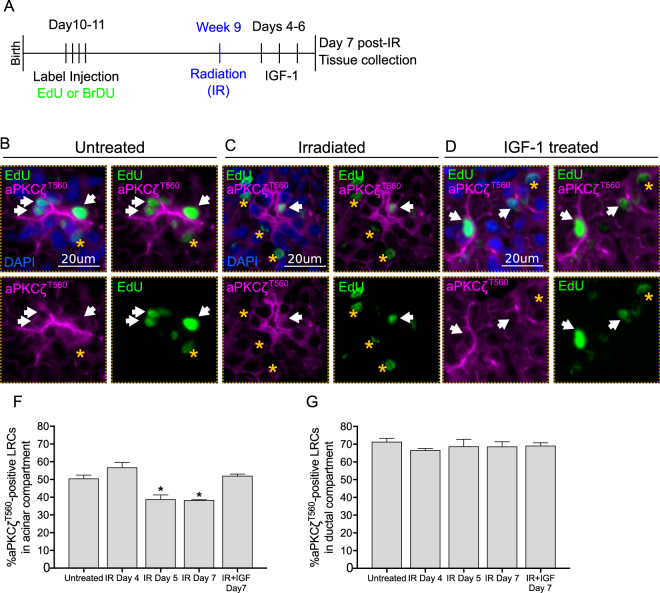
^*T560*^phosphorylation of aPKCζ in acinar LRCs is maintained during IGF-1-induced salivary gland regeneration. (**A**) Label-retaining assay was performed in FVB mice as described in methods section (n ≥ 3 for all groups). 4 days following radiation, animals were injected with 3 doses of IGF-1 (5 ug/mouse) 24 hours apart. PGs from irradiated, IGF-treated, and control mice were collected 7 days following radiation treatment. (**B**–**D**) Representative images of PGs stained for aPKCζ^*T560*^ (Magenta) and EdU (Green). White arrows point at aPKCζ^*T560*^*-*positive LRCs. aPKCζ^*T560*^*-*negative LRCs are shown with a yellow star. (**F**) Quantification of the percentage of aPKCζ^*T560*^*-*positive acinar LRCs. (**G**) Quantification of the percentage of aPKCζ^*T560*^*-*positive ductal LRCs. Star represents statistical differences vs untreated group (p < 0.05 by one-way ANOVA with follow up Tukey’s multiple comparisons test).

Quantification of LRCs that have active aPKCζ (referred to as aPKCζ^T560^), which contains a phosphorylated threonine residue at position 560^45^ was performed using immunofluorescence analysis. There is a decrease in the percentage of aPKCζ^T560^-positive acinar LRCs at days 5 and 7 following radiation (p < 0.05), but not at day 4 (p = 0.19, Fig. 4F). Administration of IGF-1 increases the percentage of aPKCζ^T560^-positive acinar LRCs compared to irradiated glands at 7 days post-treatment (p < 0.005), and levels are comparable to non-irradiated controls (p = 0.97). Interestingly, the percentage of aPKCζ^T560^-positive ductal LRCs does not change across treatments (Fig. 4G).

**Figure 5:**
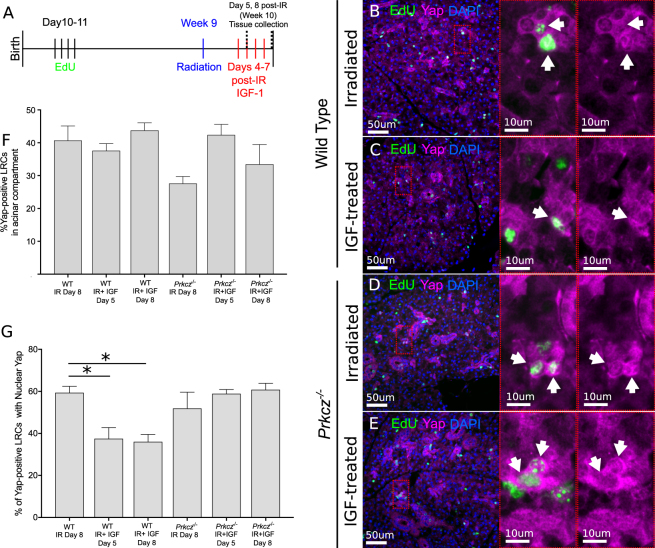
IGF-1 inhibits radiation-induced nuclear translocation of Yap in acinar LRCs. (**A**) Label-retaining assay was performed in *Prkcz*^*−/−*^ and wild-type (C57BL/6 J) mice as described in methods section (n ≥ 3 for all groups). 4 days following radiation, animals were injected with 1 or 4 doses of IGF-1 (5 ug/mouse) 24 hours apart. PGs were collected 24 hours following the last dose of IGF-1 (at day 5 or 8 following radiation). (**B**–**E**) Representative images of PGs stained for Yap (Magenta) and EdU (Green). White arrows point at Yap*-*positive LRCs with nuclear Yap. (**F**) Quantification of the percentage of Yap*-*positive acinar LRCs. (**G**) Quantification of the percentage of nuclear-Yap*-*positive acinar LRCs. Star represents statistical differences vs untreated group (p < 0.05 by one-way ANOVA with follow up Tukey’s multiple comparisons test).

Since aPKCζ regulates nuclear translocation of Yap in acinar LRCs (Fig. 3) and treatment with IGF-1 maintains phosphorylation of aPKCζ in acinar LRCs (Fig. 4), we hypothesized that IGF-1 would also modulate nuclear translocation of Yap in this population in an aPKCζ-dependent manner. To test this, wild type and *Prkcz*^*−/−*^ mice received IGF-1 at days 4–7 post-radiation and PGs were harvested 24 hours after the first and last IGF-1 injection (Fig. 5A). Consistent with previous data, the percentage of acinar LRCs with total Yap is not different across treatments (Figs 5F, S3); however, nuclear Yap is significantly reduced in WT LRCs from IGF1-treated mice when compared with irradiated animals that did not receive IGF-1 (p < 0.05, Fig. 5B,C,G; white arrows). Strikingly, the percentage of acinar LRCs with nuclear Yap remains elevated in all *Prkcz*^*−/−*^ mice, even after administration of IGF-1, and is not different from irradiated WT LRCs (Fig. 5G), indicating that IGF-1 requires aPKCζ to reduce nuclear Yap in acinar LRCs of irradiated salivary glands.

### Inactivation of aPKCζ and increased nuclear Yap in acinar LRCs persist chronically following radiation and are restored with IGF-1

**Figure 6:**
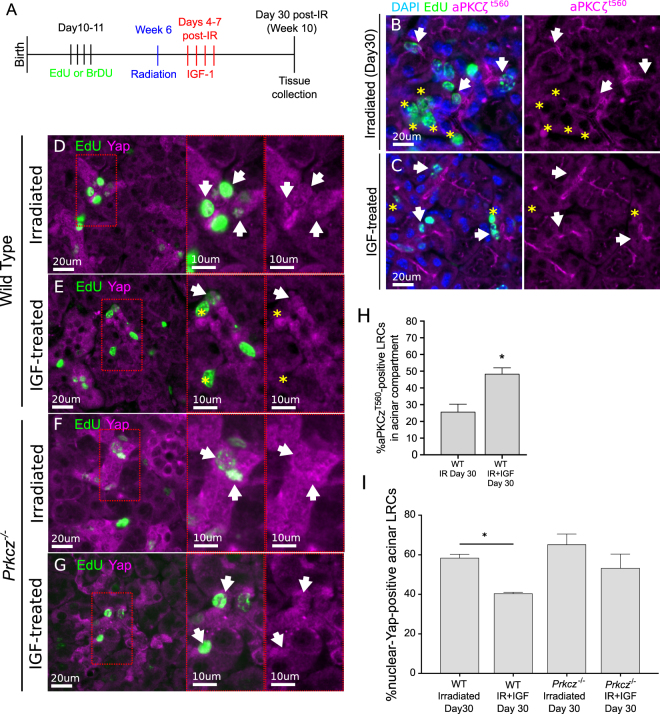
Percentage of ^*T560*^aPKCζ-positive and nuclear-Yap-positive LRCs are maintained chronically following IGF-1 treatment. (**A**) Label-retaining assay was performed in *Prkcz*^*−/−*^ and wild-type (C57BL/6 J) mice as described in methods section (3 mice were used per group). 4 days following radiation, animals were given 4 doses of IGF-1 (5 μg/mouse) 24 hours apart. PGs were collected 30 days after treatment. (**B**,**C**) Representative images of PGs stained for aPKCζ^*T560*^ (Magenta) and EdU (Green). (**D**–**G**) Representative images of PGs stained for Yap (Magenta) and EdU (Green). (**H**) Quantification of the percentage of aPKCζ^*T560*^*-*positive acinar LRCs. (**I**) Quantification of the percentage of nuclear-Yap*-*positive acinar LRCs. Star represents statistical differences vs untreated group (p < 0.05 by one-way ANOVA with follow up Tukey’s multiple comparisons test).

Multiple animal models of radiation-induced salivary dysfunction have consistently shown that hyposalivation persists several weeks to months following a single dose of radiation, which can be attributed to the loss of acinar cells and replacement by fibrotic and connective tissue^15^. To determine if activation of aPKCζ and nuclear Yap are involved in radiation-induced chronic loss of function, PGs from irradiated animals were analyzed 30 days following radiation (Fig. 6A). The percentage of aPKCζ-positive and nuclear-Yap-positive acinar LRCs at this chronic stage (30 days post-radiation, Fig. 6H,I) are comparable to those observed at day 5 (compare to Figs 3F, 4E). This suggests that inactivation of aPKCζ and increased nuclear translocation of Yap in acinar LRCs are sustained during chronic salivary dysfunction. In irradiated wild-type mice treated with IGF-1, the percentage of aPKCζ^T560^ –positive acinar LRCs is significantly higher (p < 0.05), and the percentage of nuclear-Yap + WT LRCs is significantly reduced (p < 0.05) when compared to irradiated mice that did not receive IGF-1 (Fig. 6B–I). In contrast, nuclear Yap is unchanged (p = 0.22) in irradiated *Prkcz*^*−/−*^ mice treated with IGF compared with irradiated *Prkcz*^*−/−*^ mice that did not receive IGF-1 (Fig. 6F,G,I).

### aPKCζ is required for IGF-1-induced functional restoration of irradiated salivary glands

**Figure 7:**
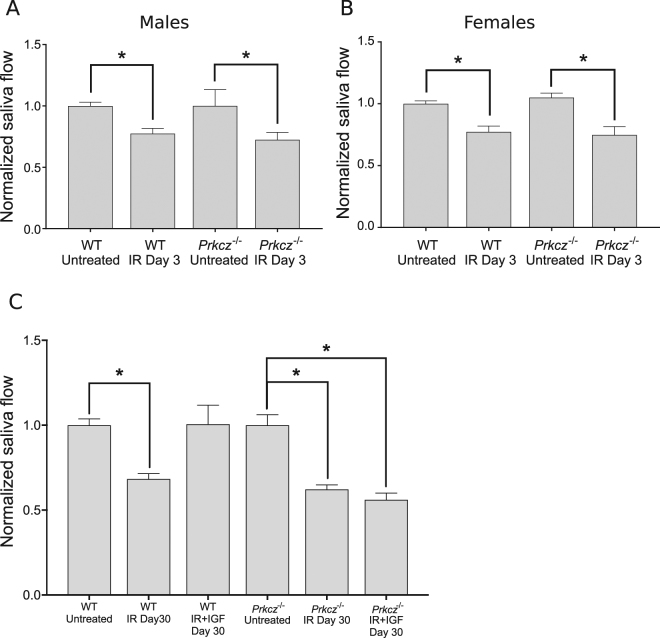
aPKCζ is required for functional restoration of irradiated salivary glands with IGF1. Saliva was collected from wild type (C57BL/6 J) and *Prkcz*^*−/−*^ mice at days 3 and 30 following radiation treatment as described in the methods section. A minimum of 9 males and 3 females were used per group for saliva collection at day 3. At day 30, at least 5 animals were used for each treatment group. Values for each mouse strain were normalized to their respective untreated control groups. Statistical differences between groups are represented by a star (p < 0.05, One-way ANOVA followed by Tukey’s multiple comparisons test).

Lastly, we aimed to determine if aPKCζ is directly involved in radiation-induced hyposalivation, as well as in functional restoration of irradiated salivary glands with IGF-1. We measured stimulated saliva output in irradiated, IGF-treated, and non-irradiated *Prkcz*^*−/−*^ mice compared to wild type controls. Acute radiation-induced loss of saliva in mice is measurable 3 days following a single 5 Gy dose of radiation, and chronic loss of function is evident by day 30 post-treatment. On the other hand, partial recovery of saliva production is accomplished 30 days post-therapy with systemic administration of IGF-1, and full recovery is observed by day 60^7^. Radiation causes acute loss of saliva in both wild type and *Prkcz*^*−/−*^ mice. In wild type animals, saliva output is reduced at day 3 post-radiation in both males and females by 22.51% and 22.71%, respectively. Similarly, saliva secretion of *Prkcz*^*−/−*^ mice is significantly decreased by 27.56% and 30.22% for males and females, respectively (Fig. 7A,B), which is not different from their wild type counterparts. At 30 days post-radiation, saliva output of irradiated wild type mice is reduced by 31.60% and 32.33% of untreated values, in wild type and *Prkcz*^*−/−*^ mice, respectively (Fig. 7C). While administration of IGF-1 induces recovery of saliva in wild type animals at 30 days post-treatment, saliva output of IGF-treated *Prkcz*^*−/−*^ mice is significantly lower compared to controls and similar to irradiated animals (Fig. 7C), indicating that aPKCζ is required for functional restoration of salivary glands with IGF-1.

## Discussion

It has been a matter of debate whether salivary gland SPCs contribute to maintain homeostasis in the adult glands and whether they can be stimulated to promote repair following radiation injury. Nevertheless, using stem cells as a potential treatment for radiation-induced salivary hypofunction and xerostomia has been suggested, primarily by means of transplantation^1,2,46–49^. Alternatively, our study suggests that the endogenous pool of salivary LRCs may be stimulated with IGF-1 to promote regeneration. However, although post-therapeutic administration of IGF-1 did not affect tumor growth rates in a xenograft model^50^, systemic administration of growth factors, such as IGF-1, raises concern of increasing the risk of developing de novo malignancies^51,52^. One way to circumvent this shortcoming is to develop a delivery mechanism, such as nanoparticles, that allows for targeted administration to the salivary glands. Alternatively, elucidating the molecular mechanisms that drive functional restoration of salivary glands upon administration of IGF-1 may provide valuable insight for the development of salivary specific and more efficient therapies. Here we show that functional restoration of irradiated salivary glands with IGF-1 requires aPKCζ and that restoration of function involves phosphorylation of aPKCζ and inhibition of nuclear Yap in acinar LRCs. Remarkably, genetic ablation of aPKCζ prevented restoration of salivary secretion upon administration of IGF-1. Our findings propose aPKCζ as a potential target for future therapies to treat loss of saliva.

During regeneration of salivary glands using the ductal ligation model, most acinar cells undergo apoptosis, yet the gland is able to regenerate from the surviving cells^53,54^. The extensive acinar cell death observed in these studies suggested that regeneration likely occurred from expansion and differentiation of a different cell type, presumably residing in the ductal system. Similarly, pioneering studies using ^3^H-Thymidine labeling assays concluded that the intercalated ducts replenished the acinar cells by a differentiation process^55,56^. It was speculated that salivary gland stem cells reside in the ducts, and that these cells are responsible for maintaining homeostasis in the adult salivary glands (See ref.^47^ for review). A more recent study challenged this point of view as it demonstrated that Mist1-positive acinar cells contribute to glandular growth, homeostasis, and regeneration through a process of self-duplication without significant input from salivary progenitors^10^. Others have also shown that most ductal cells in the adult salivary gland are non-cycling^57^, and that radiation promotes senescence in the ductal system of the salivary gland^58^. These studies may explain why compensatory proliferation post-radiation was only observed in acinar LRCs and not in the ductal system.

Radiation-induced compensatory proliferation appears to have regulatory mechanisms that are conserved across species^40,59,60^. In the *Drosophila* larval wing imaginal discs, radiation induces apoptosis and disrupts epithelial cell polarity by targeting the Par-aPKC complex, which in turn promotes proliferation of the surviving cells^21^. Similarly, stem cells in the intestine become highly proliferative in response to radiation to facilitate regeneration^40,61,62^. Genetic ablation of aPKCζ improves intestinal regeneration after radiation by increasing the proliferative activity of Lgr5+ stem cells through enhanced β-catenin and Yap signaling^40^. In contrast with observations in intestinal stem cells, genetic deletion of *Prkcz* did not potentiate radiation-induced compensatory proliferation of salivary acinar LRCs (Fig. 2B: comparison between irradiated wild type vs irradiated *Prkcz*^*−/−*^) and recovery of salivary function with IGF-1 involved an increase in aPKCζ phosphorylation in acinar LRCs. These differences might point at a unique role of aPKCζ during salivary gland regeneration. This function of aPKCζ involves inhibition of nuclear Yap without changes in the expression level of Yap. Although the specific functions of nuclear Yap in salivary LRCs remain to be elucidated, studies suggest that nuclear Yap promotes proliferation and renewal of progenitor cells, while cytoplasmic Yap is crucial for maintaining homeostasis^35^.

It is important to note that levels of nuclear Yap did not return to control levels after administration of IGF-1, even at day 30, when IGF-1 has been shown to restore salivary output^7^. Some possible explanations are that nuclear translocation of Yap is more effectively repressed before injury, or that Yap is dispensable for salivary function during homeostasis. Consistent with the latter, conditional knockout of Yap using a *Villin-Cre* system did not have any obvious defects in differentiation, proliferation, cell death, or migration along the crypt-villus axis during development^63,64^. However, intestinal regeneration was severely impaired in mice with Yap-deficient intestines following injury with dextran sodium sulfate (DSS) or radiation^63,64^. Very interestingly, loss of Yap promoted a decrease in crypt proliferation upon DSS-induced injury^63^, but induced crypt hyperplasia in the irradiated intestines^64^. Although these remarks point to seemingly opposing roles of Yap in regulating stem cell function, another study demonstrated that genetic ablation of Yap specifically in the Lgr5+ stem cells led to deficient crypt regeneration following radiation^44^, further supporting that Yap is required for proper stem-cell driven regeneration. In combination, these observations might explain our results in the salivary glands if we consider that Yap is likely to exert different functions depending on its cellular localization, as well as the influence it receives from upstream regulators such as aPKCζ. Indeed, our data suggests that a delicate balance in levels of aPKCζ phosphorylation and nuclear Yap is necessary to achieve regeneration of the salivary glands following radiation injury.

Interestingly, we did not observe compensatory proliferation or increased nuclear Yap in the submandibular glands with a single, low dose of radiation. This is not surprising given that clinical studies have shown that the threshold for recovery from radiation is 25 Gy for parotid^65^ and 39 Gy for submandibular^66^, suggesting that the parotid gland is more radiosensitive. Since the daily clinical fraction of radiation administered to salivary glands is ≤2 Gy in head and neck cancer patients, it is possible that the changes in aPKCζ and Yap occur within a few exposures of the parotid gland and changes in the submandibular gland require higher doses of radiation that occur with fractionated radiotherapy.

We propose that during salivary gland homeostasis, nuclear Yap in acinar LRCs is more effectively repressed by aPKCζ. After radiation injury, lower levels of aPKCζ^T560^ result in hyperactivation of Yap, which contributes to chronic glandular dysfunction. Finally, IGF-1 normalizes the levels of aPKCζ^T560^ while maintaining sufficient levels of nuclear Yap to promote regeneration. Future studies will be necessary to determine whether aPKCζ and Yap, or their downstream effectors, can be targeted directly in salivary progenitors to promote salivary gland regeneration. IGF-1 was administered shortly after acute loss of function developed and during a stage in which acinar LRCs initiate a compensatory proliferation program (Fig. 1), yet the effects in aPKCζ phosphorylation and nuclear Yap were sustained 30 days after treatment (Fig. 6). This suggests the existence of a therapeutic window in which LRCs can be effectively targeted to rescue the glands from radiation-induced damage.

## Methods

### Mice and label-retaining assay

Experiments in this study were conducted in both male and female FVB, C57BL/6 J, and *Prkcz*^*−/−*^ mice. For each experiment, at least 3 animals were used per treatment group. Mice were maintained and treated in agreement with protocols approved by the University of Arizona Institutional Animal Care and Use Committee (IACUC). All methods were performed in accordance with the relevant guidelines and regulations. Prkcz^*fl/fl*^ mice were generated in 129/C57BL6 mixed background by standard techniques using a targeting vector containing a neomycin selection cassette flanked by FRT sites. Flox recombination sites were introduced in introns 5′ and 3′ of Exon 10 by homologus recombination (Fig. S2A). After electroporation of the linearized targeting vector into embryonic stem (ES) cells, G418 resistant ES cells were screened for successful homologus recombination by Southern blot analyses. Heterozygous recombinant ES clones were identified (Fig. S2B) and microinjected into blastocysts from C57BL/6 J mice to generate chimeras. Germline transmitted floxed heterozygotes were selected and the neomycin cassette was removed by crossing with mice carrying the FLP recombinase. To generate global *Prkcz* knockout mice (*Prkcz*^*−/−*^), Prkcz^*fl*/+^ mice were subsequently crossed with *Sox2*-Cre to generate Prkcz^*WT/−*^ mice. *Sox2-*Cre mice were obtained from Jackson Laboratory. Heterozygous mice were interbred to generate homozygous knockouts. Absence of aPKCζ was confirmed by Western blotting (Fig. S2C).

For label retaining studies, mice were given four intraperitoneal BrdU (5-Bromo-2′-deoxyuridine, Roche, Mannheim, Germany) injections at a dose of 3 mg per 100 g of body weight, or EdU (5-ethynyl-2′-deoxyuridine, Thermo Fisher Scientific, Waltham, MA) injections at a dose of 10 mg/100 g of body weight 12 hours apart at post-natal day 10. At 10 weeks of age, mice were anesthetized via an intraperitoneal injection with Avertin (240 mg/kg, Sigma, St Louis, MO) or ketamine/xylazine (50 mg/kg/10 mg/ml) and euthanized by exsanguination for collection of the salivary glands.

### Radiation treatment

One dose of 5 Gy was administered with a ^60^Cobalt Teletherapy instrument from Atomic Energy of Canada Ltd Theratron-80. The head and neck were exposed while the rest of the body was shielded from the radiation with >6 mm thick lead to avoid systemic effects. Mice were anesthetized with an intramuscular injection of ketamine/xylazine (50 mg/kg/10 mg/ml) before radiation treatment, and were monitored until they regained consciousness. Radiation dosage calculations and maintenance of the cobalt source are conducted by the Experimental Radiation Shared Service of the Arizona Cancer Center.

### IGF-1 Injections

Mice were given a maximum of four IGF-1 doses (5 ug/mouse) via tail-vein injections 24-hours apart at days 4 to 7 after radiation treatment. For “Day 5” treatments, mice received only one injection at day 4 post-radiation and salivary glands were harvested 24 hours later. For “Day 7” treatments, mice received 3 injections, and for all other time points mice were given a total of 4 injections. Tissues were collected at the time points indicated in each experiment.

### Immunohistochemistry and Immunofluorescence staining

Salivary glands were collected and immediately fixed in 10% neutral buffered formalin (Sigma) for 24 hours at room temperature. Later, tissues were incubated in 70% ethanol and embedded in paraffin. 4 um tissue sections were obtained at the Histology Service Laboratory in the Department of Cell Biology and Anatomy at the University of Arizona and IDEXX Laboratories. Immediately before staining, tissue sections were baked at 37 °C for 20 minutes to fully adhere tissues to glass slides. Tissues were then rehydrated in Histo-clear (National Diagnostics, Atlanta, GA), graded ethanol (100–50%) and distilled water, by performing 2 sequential washes in each solution for 5 minutes. A permeabilization step in 0.2% TritonX (MP Biomedicals, Santa Ana, CA) and 0.05% Tween20 (Thermo Fisher Scientific) in 1 × PBS was performed for 15 minutes prior to antigen retrieval, which was done by boiling samples in microwave for 10 minutes in 1 mM citric acid buffer (pH 6.0). Slides were left in citric acid buffer for additional 20 minutes to cool down. Non-specific binding sites were blocked with 300 ul of 0.5% NEN (Perkin Elmer, Waltham MA). EdU staining was performed according to the manufacturer’s protocol (Click-iT® Plus EdU Alexa Fluor® 488 Imaging Kit, Thermo Fisher Scientific). Tissues were incubated in primary antibody overnight at 4 °C followed by incubation in secondary antibody for 1 hour at room temperature. Both antibodies were diluted in 1%BSA (Sigma) in 1 ×PBS. Lastly, a nuclear stain with DAPI (1 ug/mL) was performed in the dark for 3 minutes and slides were then mounted with a solution of ProLong® Diamond Antifade Mountant (Thermo Fisher Scientific). Slides were stored at 4 °C overnight before imaging with a Leica DM5500 microscope (Leica Microsystems, Wetzlar, Germany) and an ORCA-Flash4.0 LT Digital CMOS camera (Hamamatsu Photonics K.K., Japan). We used primary antibodies anti-Ki67 (1:200, #12202), and Yap1 (1:250, #14074) purchased from Cell Signaling (Danvers, MA); total aPKCz (1:100, #10860-1-AP) from Proteintech (Rosemont, IL), and anti-phospho-aPKCζ^T560^ (1:250, Abcam, Cambridge, UK. ab62372). Analysis for each target was performed by manually counting positive cells from a minimum of 5 fields of view using a 40× objective. Two 4 um sections from a minimum of three slides (three mice) per group were analyzed. For analysis of Yap-positive area, we used the threshold function of Fiji. During analysis, we deemed the ductal compartment as everything that could be identifiable as a duct based on morphological features only, such as a rounded structure, the presence of a lumen, and tight cell-cell contacts. This compartment includes the excretory and striated ducts, as well as some of the intercalated ducts. Thus, the acinar compartment comprises all the remaining cell types in the salivary epithelium: mainly acinar and myoepithelial cells, as well as intercalated ducts that based on morphology could not be identifiable as ducts. Acinar and ducal compartments were analyzed individually and statistics were performed as described in the statistical analysis section.

### Saliva collection

Stimulated saliva was collected from C57BL/6 J and *Prkcz*^*−/−*^ mice at 3 and 30 days following radiation treatment. A minimum of 5 mice were used per group except for female Prkcz^*−/−*^ mice at day 3, which has an n = 3. Mice were given a 0.25 mg/kg dose of carbachol (Sigma-Aldrich) immediately before collection. During collection, mice were restrained, and saliva was collected in pre-weighted tubes via vacuum aspiration for 5 minutes. Milligrams of saliva were calculated by substracting the weight of the empty tubes. Saliva flow per minute was calculated for analysis and normalized to wild type unirradiated values. Saliva samples were immediately placed in dry ice and further stored at −80 °C.

### Western Blot

Parotid glands from wild type and *Prkcz*^*−/−*^ mice were lysed in RIPA buffer with 5 mM sodium orthovanadate (Fisher Scientific, Waltham, MA), protease inhibitor cocktail (Sigma-Aldrich, St. Louis, MO) and 100 mM PMSF (Pierce/Thermo Scientific, Rockford, IL); then, samples were sonicated for 2 minutes and boiled for 10 min until homogeneous. 100 ug of protein from supernatant were loaded in 12% polyacrylamide gels. Transference was performed for 1 hour at 100 V using 0.45 μm Immobilon-P membranes (Millipore, Billerica, MA). After blocking in TBST buffer containing 2% BSA, membranes were incubated at 4 °C overnight in primary antibody at 1:1000 dilution. For detection, HRP conjugated secondary antibodies were applied to the membranes for 1 hour at room temperature and ECL substrate (Pierce/Thermo Scientific) was used following instructions by the manufacturer. The following antibodies were used: anti-total aPKCζ (Cell signaling, #9368), anti-PKCζ (phospho T560) antibody [EP2037AY] (Abcam, ab62372), Anti-Beta-tubulin (Thermo Scientific, #rb-9249-p), anti-Yap (D8H1X) XP Rabbit mAb (Cell Signaling, # 14074), anti-Rabbit-HRP (1:2000, Cell signaling, #7074 S), and Goat-anti-rabbit (1:10000, BioRad, #972-4446).

### RT/PCR

RNA was isolated from PG using Qiagen RNeasy Mini Kit. cDNA was prepared with SuperScript IV Reverse Transcriptase (Thermo Scientific, #18091050) following the manufacturers instructions. RT-PCR was performed with 10 ng of cDNA and the following program: 1 cycle at 95 °C for 15 min; 40 cycles at 95 °C for 15 sec, 54 °C for 30 sec, and 72 °C for 30 sec; and then kept at 4 °C. All primers were purchased from Integrated DNA Technologies (Coralville, IA). Primer sequences are as follows: *Yap* (Forward: TGT GCT GGG ATT GAT ATT CCG TA Reverse: ACC CTC GTT TTG CCA TGA AC), *Gapdh* (Forward: ACC ACA GTC CAT GCC ATC AC, Reverse: CAC CAC CCT GTT GCT GTA GCC), *Prkcz* (Forward: CAG GGA CGA AGT GCT CAT CA, Reverse: CAC GGC CGG TAG ATG GAC TTG).

### Data analysis

Statistical analysis and graphing was performed using Graph-Pad software (version 7.01, La Jolla, CA). Cell counts from immunofluorescence stains were analyzed by a one-way analysis of variance (ANOVA), followed by Tukey’s multiple comparisons test. RT-PCR data is normalized to GAPDH loading controls and expressed as fold-change vs wild type levels for each gene. Salivary flow rates were normalized to unirradiated wild type controls and analyzed by one-way ANOVA followed by Tukey’s multiple comparisons test.

## Electronic supplementary material

Supplementary Information
