# Supplementary material for: aPKCζ-dependent Repression of Yap is Necessary for Functional Restoration of Irradiated Salivary Glands with IGF-1
Source: Sci Rep. 2018 Apr 20;8:6347. doi: 10.1038/s41598-018-24678-4 (PMC5910385; doi:10.1038/s41598-018-24678-4)
Supplement: Supplementary file 1 — Supplementary Information [file 41598_2018_24678_MOESM1_ESM.pdf]

# Supplementary Information

# aPKCζ-dependent repression of Yap is necessary for Functional Restoration of Irradiated Salivary Glands with IGF-1

Alejandro M. Chibly, Wen Yu Wong, Maricela Pier, Hongqiang Cheng, Yongxin Mu, Ju Chen, Sourav Ghosh, and Kirsten H. Limesand

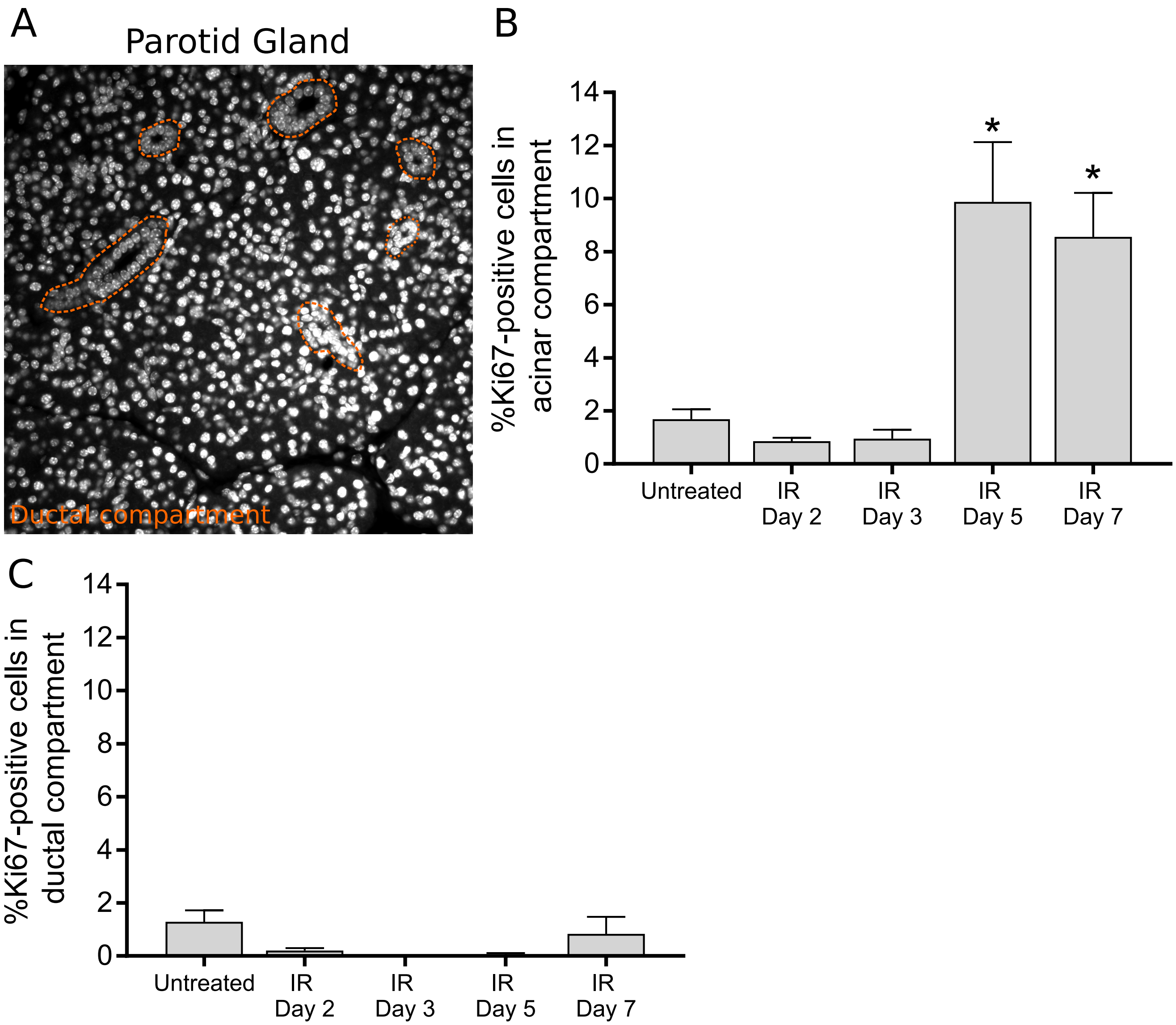


**Figure S1.** Acinar and ductal compartments of the gland were analyzed individually as described in the methods section. Ductal compartment is shown in the delineated areas with a dotted line in panel A). All remaining cells were considered part of the acinar compartment. B) Quantification of the percentage of Ki67+ (proliferative) cells in the acinar compartment. C) Quantification of the percentage of Ki67+ cells in the ductal compartment. Star represents statistical differences vs the Untreated group ($p<0.05$) by one-way ANOVA followed by Tukey's multiple comparisons test.

.

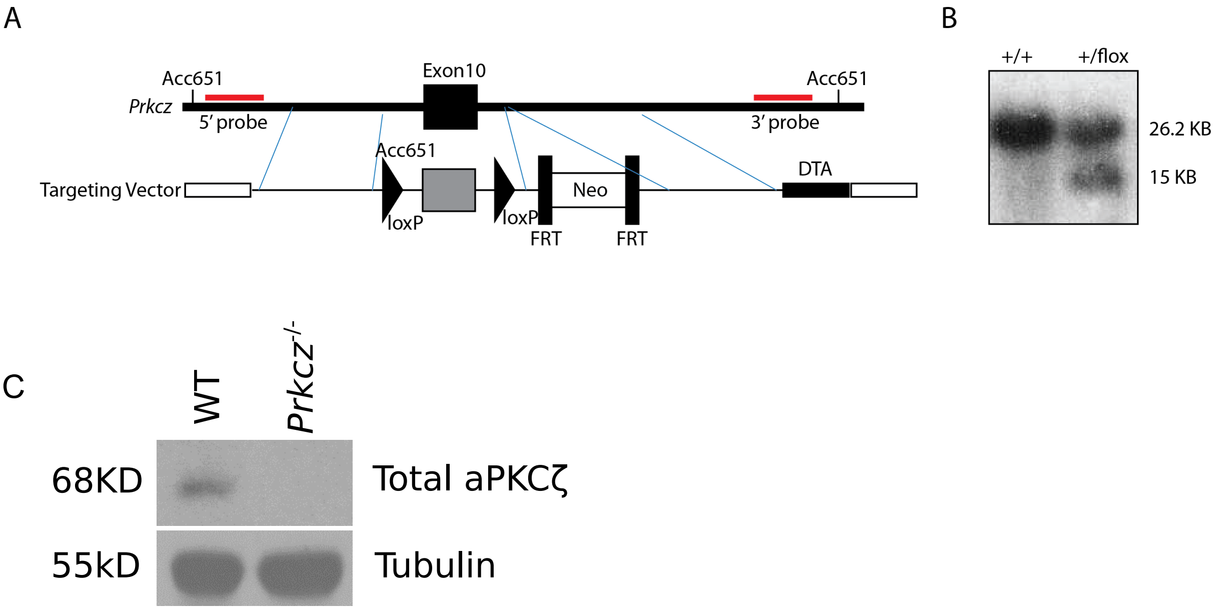


**Fig S2**. A) Cloning strategy for the generation of *Prkcz*-floxed mice. B) Identification of the floxed allele by southern blotting. *Prkcz*$^{fl/fl}$ mice were subsequently crossed with Sox2-Cre to generate *Prkcz*$^{-/-}$ mice. C) Western blot confirmation of aPKCζ depletion in parotid glands from adult mice.

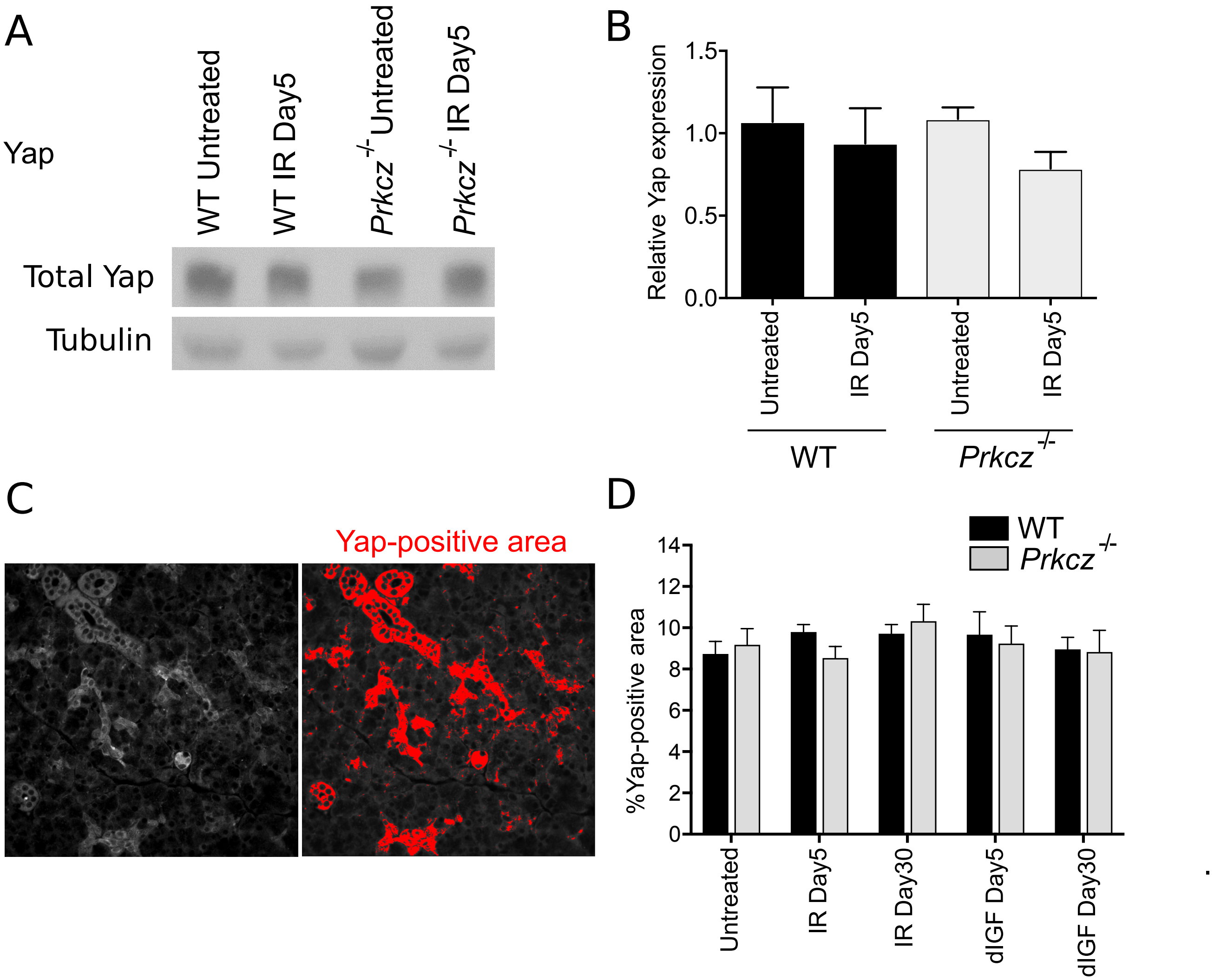


**Fig S3**. A) Western blot of whole parotid homogeates from WT and *Prkcz*$^{-/-}$ mice. Tubulin was used as loading control B) Relative gene expression of Yap in cDNA from whole Parotid lysates of irradiated and control WT and *Prkcz*$^{-/-}$ mice.
C-D) Representative Yap staining and thresholding funcion of Fiji used to quantify percentage of Yap-positive area. Quantification is shown in D. E) Western blot of whole parotid homogenates for total and active aPKCζ. F) PCR of whole parotid from WT mice for *Prkcz*.

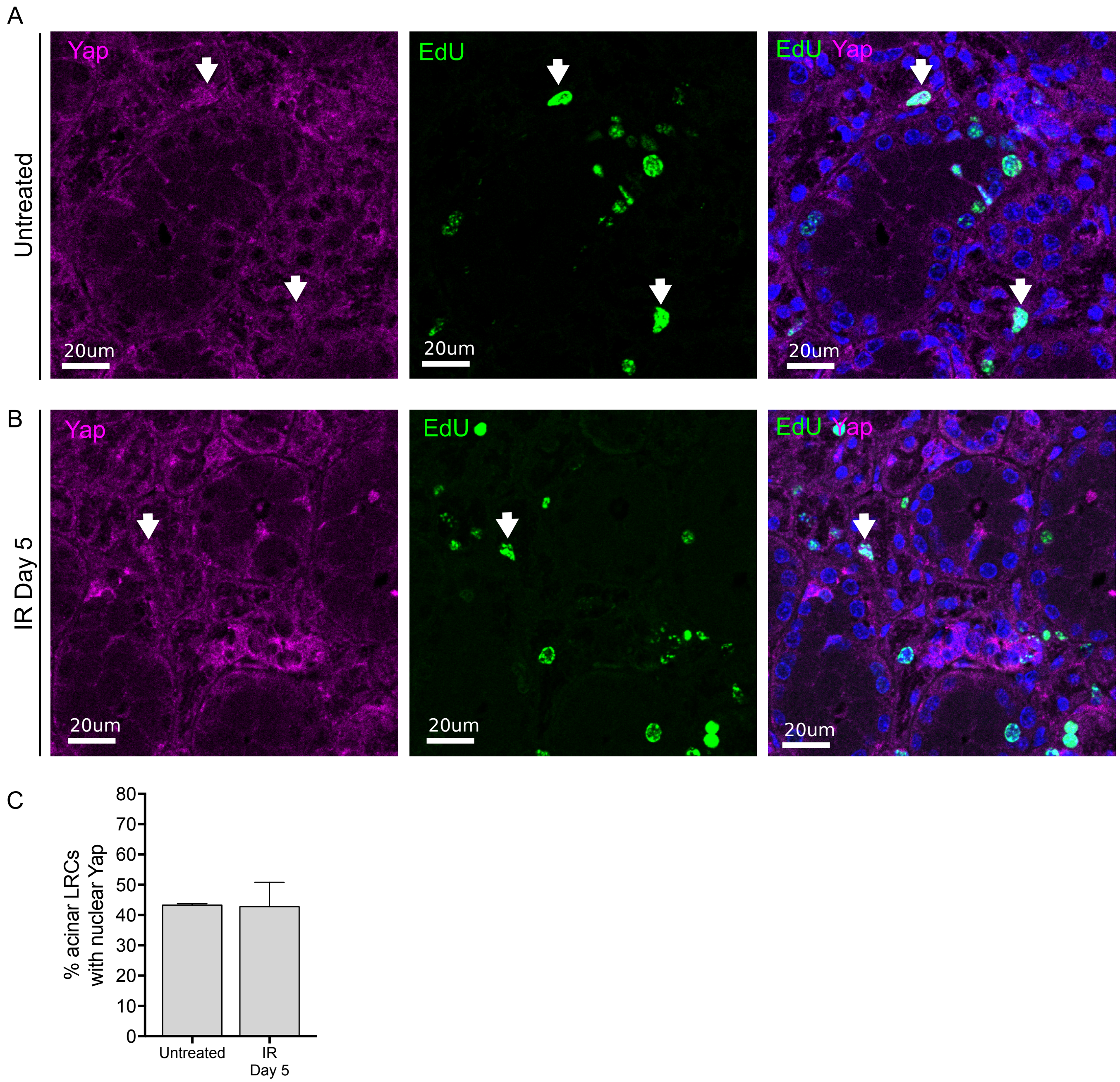


**Fig S4**. Representative images of control (A) and irradiated (B) SMG stained for Yap, EdU and DAPI. White arrows point at LRCs positive for nuclear Yap. Quantification is shown in panel C.
